# Clinical Report of Stray Cases of Constitutional Syphilis

**Published:** 1873-07

**Authors:** T. Curtis Smith

**Affiliations:** Ohio


### CLINICAL REPORT OF STRAY CASES OF CONSTITUTIONAL SYPHILIS.

BY T. CURTIS SMITH, M.D., OF OHIO.

The following cases being of more than usual interest to me, I thought they might possibly prove interesting to other members of the profession:

Mrs. A., æt. twenty-eight, the mother of four children, youngest æt. eighteen months. She is of a lymphatic temperament; had been sick for six months, during which she had been treated for phthisis pulmonalis, which she and her friends believed she had. At the time I first saw her, January 10th, 1872, I found her in bed, pale, anemic, emaciated; pulse one hundred and twenty, feeble; respirations twenty-four per minute; expression anxious; no headache, but considerable vertigo; is unable to sit up or lay entirely down, but reclines on pillows; tongue furred, bitter taste; appetite very poor; breath fetid; mucous membrane of pharynx congested and sore; has frequent dry, hacking cough; sputa white, frothy and tenacious; voice hoarse and feeble; respiratory sounds clear and somewhat exaggerated all over both lungs; more than usual resonance on percussion, probably owing to the attenuated condition of the chest walls; could find no evidence of tubercular deposits or of the existence of cavities, though she had twice had slight hemoptysis. Was now satisfied that her disease was not phthisis pulmonalis. The stomach and bowels not seriously deranged, though very costive, not having an evacuation without using physic; the liver of normal size and giving no evidence of disease; urine normal and of usual quantity. The uterus was somewhat enlarged and congested, with granular cervicitis existing; there is, also, quite profuse muco-purulent leucorrhœa; some tenderness of the cervix; heat of vagina normal; under axilla 96° F. The post cervical, inguinal glands and Sigmund's glands I found *enlarged.* The former she stated had been so for at least a year. All questions as to having suffered from specific disease were pointedly denied. Now to a further point of interest in the case. Eleven months after confinement she menstruated once, but not since that date. A month later, had slight hemoptysis; and three weeks later it occurred again, after which time epistaxis occurred every three or four weeks, lasting from one to four days; not severe, but nearly continuous for that period. I find the left nostril quite closed with a thick and black incrustation; the bleeding nearly always occurring from this left side; the mucous membrane of the right nares congested. These attacks of nasal hemorrhage always brought on severe prostration. Diagnosis: Real disease, constitutional syphilis. Knowing a little of the ten thousand forms assumed by this loathsome disease, I believed from the history and symptoms of the case, and knowing the irregular habits of the head of the family, that my diagnosis was correct beyond a doubt, though it might not seem so plain to others.

In this case, I think, the syphilitic poison first produced disassimilation, through its influence over the glandular system; out of this resulted first, an impoverished condition of the blood, and finally, anemia and emaciation, and this condition favored the derangement of the menstrual functions and local congestions, such as were observed in the mucous membrane of the pharynx and nares; hence, the free discharge of blood from the left nares, where a solution of continuity existed, from ulceration of the schneiderian membrane. Besides the mere fact of the existence of such local congestion in this particular case, we know the schneiderian membrane and the pharynx are very liable to be affected in constitutional syphilis. The two attacks were probably produced by a determination of blood to the lungs, favored by the conditions above named, and to my mind, seemed to take the place of proper menstruation, as did, also, the epistaxis.

*Prognosis.*—Rather grave, but not despairing.

*Treatment.*—Placed her on the following: ℞. Hydr. proto. iod., grs. viii.; ferri pulvis, quinia sulphs., aa. grs. xxxii.; strychnia sulph., grs. ii. Mix. Fiat pilulæ, xxxii. S. One every four hours through the day for four doses Also: ℞. Ext. hyoscyam., ℈iss.; pulv. aloes, cape., grs. xv. M. ft. pil. xv. S. One at bedtime and one each morning.

She was also to have egg-nog or milk-punch liberally; also, beef essence, oysters; in short, to have a good, nourishing diet, selecting such articles as she could best digest. Friction to the entire surface every morning with a crash towel wrung out of strong tepid mustard or salt-water. Good hygienic measures were rigidly carried out, and everything kept up as cheerful as possible.

Topically, brown mercurial ointment was applied to the intra-cervical disease every few days and every night; a mercurial vaginal suppository inserted and strict cleanliness required. The cervix under this management soon became healthy in appearance, and the leucorrhœal discharge ceased. She was kept on the above treatment with little apparent change for better or worse, except in the points above named, for three months. After about that length of time improvement was quite apparent, but slow. The epistaxis failed to return after the tenth week, and nares assumed a healthy appearance; the cough ceased; appetite improved; bowels became regular without aid; patient cheerful. At the end of fourteen weeks she could sit up, and soon walked about her room or rode out. At four months from commencing treatment menstruation appeared, quite normally, and has continued to appear regularly ever since. The same treatment was continued two months longer, after which it was gradually relinquished. After six months of continuous, steady treatment on this plan, I had the pleasure of seeing her look and feel hearty and well. I believe my diagnosis was correct, and that the treatment proved it to be so, otherwise I shall be forced to the conclusion that the patient recovered in spite of an incorrect diagnosis, and an incorrect plan of treatment. In the light of a diagnosis establishing any other disease, with a patient thus debilitated and thoroughly emaciated, how could a man be justified in giving mercury continuously for six months, without intermission? Take a case reduced to this low point from any other cause, and it would be very doubtful whether she would ever recover with a fourth of a grain proto. iod. hydr. poured into her stomach several times a day, whatever else the treatment might be.

*Case* 2. In July, 1871, Mrs. L., æt. twenty-one, came to my office for treatment; stated that she had been in good health, though not robust, till about a year after marriage, when she ceased to menstruate; was attacked with rheumatic pains in the extremities; became weak and debilitated; lost her appetite; became very costive; slept but little. This condition begun about January of same year. I find her pulse one hundred, feeble; temperature ninety-seven and a-half; skin dry and furfuraceous; no glandular enlargement; no leucorrhœa; not pregnant; uterus of normal size, not diseased in any way. Lungs, liver, kidneys, stomach and bowels free from any perceptible organic disease. Catamenia absent since January (about the) 4th. I looked for evidences of syphilis, but did not find any; there was no lesion, nor was she aware that there ever had been. She had never been *enciente.* Here I thought must be a case of general debility, nothing more. I evacuated the bowels with a vegetable cathartic, and to keep them regular, gave same pill of aloes and hyoscyamus as in the other case. I also gave: ℞. Tr. columbœ, ℥iii.; spts. frumenti, ℥ii.; tr. cardamon, ℥i.; syr. simplex, ℥ii.; ferri sulp., grs. vi. Mix. Sig. Tablespoonful after each meal. To have good nourishing diet, and avoid any over-exertion. This failed for good, except to regulate the bowels. I wrung the changes on tonics for two months, without apparent avail. Again I searched for the cause of her condition, as I had done several times before. I found but one post cervical gland enlarged very slightly, and very slight enlargement of two inguinal glands. No nodes, but many "wandering pains" through limbs and back. I now placed my patient on the following: ℞. Potass. iodid., ʒvi.; hydr. bichloridi, grs. ii.; syr. sarsaparilla comp., ℥viii. Mix. Sig. Half tablespoonful after each meal. When this was gone she returned, stating she was better. Treatment continued. After two months she menstruated, and has been quite well ever since. The symptomatic evidence of constitutional syphilis in this case was quite imperceptible until the effect of the last above-named agents were brought to bear, when I think it became clear that such disease existed, though I admit that such could not be accepted as undeniable proof of its existence.

Early in 1872, Mrs. ——, white, æt. 26, came asking treatment; had been married five years; mother of three children, youngest sixteen months old. Had for past six months suffered much from vertigo and pain in the head, and occasionally had epileptoid convulsions; had never been otherwise than stout and robust till after the birth of the last child, since which she had never felt well. At time of coming to me the right side is partially paralyzed and choreaic; menstruation irregular for last six months, at which time it first appeared after birth of last child; leucorrhœa slight. There was evident anemia from the pallor; quick, feeble pulse, etc.; but the leading symptoms were the hemiplegia and epileptoid attacks. There was no history of epilepsy or paralysis, or of apoplexy in her family. She had always been well up to a stated time; these more serious symptoms came on suddenly and without warning. Reasoning by exclusion, with the case before me, I somewhat indefinitely arrived at the conclusion that this was a case of hemiplegia and epilepsy, with syphilis as the exciting constitutional cause. I had never seen the husband, and but one of the children, which I thought showed a few marks of congenital syphilis. But the mere fact of the epilepsy coming on suddenly at that age, in a previously healthy constitution, and of its accompanying hemiplegia, without evidence of cerebral hemorrhage, abscess or injury, and without any perceptible trace of spinal disease, was very strong presumptive evidence of its being caused by syphilitic infection. So much so, that I think in the majority of such cases a diagnosis can tolerably safely rest upon this evidence alone, though of course it is wise to bring all the testimony to bear in every case. I placed this patient on strong antisyphilitic and tonic treatment, and after the first week had the pleasure of seeing her commence a rapid recovery, which has seemed to be quite perfect. Months after the treatment had closed, I became acquainted with this lady's husband, who was suffering from syphilitic manifestations too plain to be mistaken, and of a tertiary nature. This circumstance and the lady's recovery on the plan above named, were strongly confirmative of the correctness of the diagnosis in her case.

Many more such cases of obscure or entirely negative evidence of constitutional syphilis being present could be enumerated, but we deem it a needless task, and that it would give this article an undue length. Therefore I shall only casually mention a few cases in my further remarks.

To recognize primary and many forms of secondary and tertiary syphilis is quite an easy task. But to be always cognizant of its presence when marked with the semblance of other diseases or pathological conditions is not always so easy. No disease can put on more multiplied forms than this. It may simulate any thing from a mere ungual brittleness and all forms of light or serious skin diseases, up to the most grave forms of spinal, cerebral or visceral lesions, and, as Prescott Hewit recently stated, (in substance) it is the more difficult to recognize from the fact that often no syphilitic history can be obtained, the peculiar manner in which it manifests and the great lapse of time that often intervenes between the different stages. He has related cases more strikingly obscure probably than those mentioned in this paper, but, yet, evidently cases of syphilis. (*The Clinic,* Vol. iv., No. 12., from the British *Medical Journal,* February 22, 1873.) A few years of practice and close attention to the freaks of this disease will suffice to show its manifold shapes, many of which it is impossible to find described in special works on specific diseases.

Three years ago I was called to see a man suffering severely from a large chronic ulcer on his leg. He had been in good hands for some months, but had failed to be cured. I know, positively, that he had contracted syphilis some years before. I placed him on antisyphilitic agents, and in a short time he was well and at work, and that without any special local treatment. Several such cases have been relieved quickly by a knowledge of their constitutional taint.

Only a month since a young man came complaining of pain in the cardiac region. I know him to be a syphilitic. Examination showed valvular lesions of left side of the heart, and what seemed to be chronic endocarditis. A short mercurial course has greatly improved his condition. Where such a knowledge of infection is not possessed by the physician, he will often be left entirely in the dark, unless some stray, yet quite marked symptoms of the disease are found.

It is almost useless, often worse than useless, to ask the patient if he has ever been infected, as most men and women are foolish enough to deny such facts, much to their own detriment. Some years since a man came complaining of a swelled and very painful testicle. I thought it was caused by syphilitic infection, but there was no very clear evidence, as I thought (for I was then verdant in syphilitic symptoms and pathology). He firmly denied any infection as ever having occurred with him. I thereupon gave him such treatment as is usually commended for such cases, when not specific in character. No benefit resulted, and in a short time the case slipped from my fingers into those of the man who treated him for primary sore. He then soon recovered. Several cases of different forms served to open my eyes to a deeper study and more careful search into the nature of such odd cases. Many and multiplied do I find the manifestations, which can often be learned no where but at the bedside or in the office. It is easy to discover mucus patches; the characteristic sore throat, enlarged post cervical glands; nodes; nocturnal pains, and the nature of syphilides; but when a syphilitic subject comes to us with whose history we have no acquaintance, and he denies to us that he has been infected, it is not so easy to decide whether some of his obscure troubles have their origin in a lesion that is constitutional and beyond positive observation. How shall such cases be detected? I willingly acknowledge my inability to portray the many little points by which we will be aided in a diagnosis.

Two years ago I was called hastily to see a child a few months old, the patient of an old and able physician. I found it struggling for breath; pale; pulse quick and feeble; and the nares blocked up with a cheesy-looking matter. I was at first puzzled, for it was in a family where syphilis in any form would not be expected. I soon recognized that the Schneiderian membrane was the seat of the exudation. This roused my suspicion to further search, when I discovered a pleiades of enlarged glands about its neck. I prescribed for temporary relief, but was curious to know what was the treatment by the family physician, and was not the least surprised to learn that it was purely antisyphilitic. A year and a half later I was called to see the next older child in the same family, which was suffering from a very deceptive form of cerebro-spinal meningitis. So much so, that with the greatest care I was, till near the close, undecided whether I had that grave disease to manage or not. But remembering my former experience with the family, I gave the child mercury quite freely as a cathartic, and in small doses afterwards, with nervine agents. On the morning of the third day after the attack the child was seized with an oposthotonic convulsion, in which it died an hour later. The younger child was then seized precisely as had been the other, and with the same deceptive symptoms, and so deceptive was it, and apparently so mild, that one quite able physician stated in consultation that if the case was his "without the light of the case already dead, he would tell them to put it in bed, give it plenty of cold water to drink and would expect to find it well to-morrow." This case the old family physician treated. Its age, two years, and he gave it hydr. chlor. mite, grs. vii., every three hours for three doses, following it with oil; after which he gave it half a grain of the same agent every hour for twenty-four hours. The child recovered. The case I treated might or might not have recovered; but if I had given it such heroic doses of calomel, and its death had occurred, I would have condemned my treatment as much then as I now do the lighter dosing in that particular case. It would be hard to convince that old physician that it was not the antisyphilitic properties of the agent in this particular instance, that saved the second case. Whether he is correct in this view many will question, and both he (D. C. Rathburn, M.D., of this place) and the others of us in council expressed a doubt of the special effiicacy of mercury in this disease, when not influenced by syphilitic poison. True, the case might have recovered without any treatment. So can we reason of many cases of this form of disease; but I think it is a good example of the advantage of quick and powerful antisyphilitic treatment and of a physician's knowledge, from experience, of how much a certain case will bear.

During last summer a stout, healthy-looking young man requested treatment for rheumatic pains of the right shoulder, elbow and knee. Examination showed no swelling, redness or tenderness about the joints; and they could be moved and handled freely without any increase of pain. The suffering was usually greatest at night, which is the case with both rheumatism and syphilitic pains. I gave him alkaline and anodyne treatment; then acid and anodyne; tried colchicum and guaiac and various other agents, without any considerable relief. I surmised infection by syphilis from the first, but was determined to prove, or not prove its existence by the effect of therapeutic agents. I had not treated him before and thought him above suspicion up to that time. There was not a trace of a symptom of syphilis, except these pains. At last I placed him on kali. iodat., with hydr. bichloridi and syr. sarsaparilla. He uncorked the bottle, took a draught of it, gave me a knowing squint, saying: "Golly, Doc., that tastes familiar; that'l knock it!" and, sure enough, it did almost as by magic.

Last autumn a young man, of high standing, came complaining of his throat, said he had tried all the doctors wherever he had been and could only get a very temporary relief from the soreness. I learned from him various plans of treatment and agents taken, which I knew were celebrated in the treatment of throat inflammations. I then examined his throat carefully and could observe what would most always be taken for folicular pharyngitis. But I observed several deep pits with induration around them. I could find no further trace of infection in any shape. But as others had failed, I thought I could do no worse, even if I was mistaken as to its nature. So I placed him on the prescription named above, with the most perfect success as to his relief. He, also, gave me to understand that he had occasion to use similar agents before.

In this article I have not attempted any depth of the pathology of the disease in question; but simply to bring forward a few, out of a great many cases that are continually coming under the observation of every practitioner in village, town or city practice, and to show that experience teaches that it will do no harm to be on the constant lookout for this ugly serpent of so many phases. None are too high for us to suspect of it on reasonable evidence; nor any too low for it to be found occasionally in their veins. I am satisfied that I have, and that others also, within the scope of my observation, have let cases go on from bad to worse, not suspecting the real disease, because it assumed the shape of some malady more tangible in form. I am quite firm in the belief that when the system has once become saturated with syphilitic poison, that no course of treatment, however varied and thorough, will so completely remove it from that system that it may not return again, though a score or even two score of years may intervene between the times of its clear manifestations. Hewitt (the *Clinic, loc cit*) mentions one case where twenty-eight years intervened; and another distinguished English authority, whose name I can not now recall, gave another instance where after an interval of forty-five years, its manifestations were unmistakable. A case recently came under my notice where the patient stated that I must be wrong in my opinion as he had not seen a sign of the disease for sixteen years. But he had nodes and noctural neuralgia too well marked to admit of a mistake.

I have only indicated a rough outline of treatment for syphilis, and one might suppose mercury and kali. iodat. the only agents to which I resort. Such is not the case, however, though these are certainly the standard agents to be relied on, while in an exceptional case, now and then met with, where too much mercury has already been used, the disease will yield to a tonic and vegetable alterative course. From what I have already stated, I mean that it should be remembered by us all that, *if we know a patient has been infected, that whatever else he may have in the future in the way of disease,* we should by no means forget the fact that *that infection, however remote, may exert a strong influence for evil in a disguised form; and knowing this, we should be able to meet it with proper treatment at all points.*

